# Impact of Limb Salvage on Prognosis of Patients Diagnosed With Extremity Bone and Soft Tissue Sarcomas

**DOI:** 10.3389/fonc.2022.873323

**Published:** 2022-06-06

**Authors:** Kaixu Yu, Ying Chen, Kehan Song, Fanxiu Xiong, Yahao Tian, Hanfeng Guan, Feng Li

**Affiliations:** ^1^Department of Orthopedics, Tongji Hospital, Tongji Medical College, Huazhong University of Science and Technology, Wuhan, China; ^2^Department of Obstetrics and Gynecology, Tongji Hospital, Tongji Medical College, Huazhong University of Science and Technology, Wuhan, China; ^3^Department of Epidemiology and Biostatistics, University of California, San Francisco, San Francisco, CA, United States

**Keywords:** limb salvage, amputation, sarcomas, survival, metastasis

## Abstract

**Background:**

Although clinicians and patients with extremity bone and soft tissue (EBST) are increasingly interested in limb salvage surgery (LSS), because of the minimal damage to physical appearance and function, however, there is still a lack of large-scale population studies on whether LSS improves the prognosis of patients.

**Purpose:**

The aim of this study was to compare the survival of patients with EBST sarcomas after receiving LSS and amputation.

**Methods:**

To conduct the population-based study, we identified 6,717 patients with a histologically diagnosed bone sarcoma and 24,378 patients with a histologically diagnosed soft tissue sarcoma from the Surveillance, Epidemiology, and End Results database. We analyzed overall survival (OS), cancer-specific survival (CSS), and non-sarcoma survival (NSS) using the Kaplan–Meier method, log-rank test or Gray test, Cox regression model, propensity score-matched analysis, and landmark analysis.

**Results:**

LSS could improve the prognosis in patients with most EBST subtypes, except for Ewing sarcomas and MPNST. However, in the subgroup without distant metastases, limb salvage increased CSS only for patients with osteosarcoma, Ewing sarcoma, and leiomyosarcoma, as well as NSS for patients with chondrosarcoma and synovial sarcoma. Landmark analysis further demonstrated that sarcoma survivors surviving <10 years could benefit from LSS but not for long-term survivors ≥10 years. Moreover, for patients with distant metastases, LSS could improve survival of osteosarcoma patients but worsen CSS among patients with MPNST. Landmark analysis further demonstrated that LSS improved survival among osteosarcomas patients with distant metastases only within 1 year after surgery. Moreover, patients receiving LSS and those receiving amputation had a high risk of dying from different non-sarcoma diseases during the postoperative follow-up.

**Conclusions:**

The impact of limb salvage on the prognosis of patients depends on the pathological subtype and stage of EBST sarcomas.

## Introduction

Although extremity bone and soft tissue (EBST) sarcomas comprised a collection of rare malignant tumors that arise from mesenchymal tissue (1, 2), they were responsible for more deaths than testicular cancer, Hodgkin’s disease, and thyroid cancer combined due to their more recurrent and metastatic nature (3). Historically, amputation has been the primary treatment of sarcoma, but since the 1980s, limb salvage has been considered a clinically acceptable treatment for the local control of EBST sarcomas, which is attributed to the improved imaging techniques and adjuvant chemotherapy (4, 5). However, in some cases, such as distant metastases of sarcomas, amputation remains an effective option (6, 7).

Despite the conventional wisdom that radical surgery reduces recurrence rate and complications (8), limb salvage surgery (LSS) has become increasingly attractive to orthopedists and patients with EBST sarcomas because of the minimal impairment to physical appearance and function. However, there remained a question as to whether LSS has a detrimental effect on the survival of sarcoma patients (9, 10). In a comparative study encompassing 1,220 patients with osteosarcoma treated with neoadjuvant chemotherapy, 36% and 20% of patients developed local recurrence following limb salvage with intralesional margins and marginal margins, respectively, but no events were observed among patients receiving amputation (11). Depending on the severity of recurrence and complications after LSS, patients may potentially need to undergo a secondary amputation, which could result in a poor prognosis compared with patients receiving primary limb salvage or primary amputation (12, 13). However, using the National Cancer Database, Daniel et al. analyzed the outcomes of 2,442 patients with primary osteosarcoma in the United States, including 1,855 patients receiving LSS and 587 patients receiving amputation, and reported a significant survival advantage for LSS compared to amputation (14). Moreover, no difference in overall survival (OS) was observed among patients with soft tissue sarcomas, as reported by Mavrogenis et al. (15) and Alamanda et al. (16).

Previous studies on the use of limb salvage among patients with EBST sarcomas were limited by small sample sizes and data collected mostly at single institutions (10, 14, 17, 18). There are few retrospective studies with results to inform clinical practice. To address this gap, we conducted the large population-based study using data from the Surveillance, Epidemiology, and End Results (SEER) database. First, we analyzed recent trends in the incidence of LSS among patients with EBST sarcomas. Second, we aimed to identify the characteristics of patients who were more likely to receive LSS. Finally, we sought to compare the survival of patients who underwent LSS with those who underwent amputation.

## Patients and Methods

**Figure 1:**
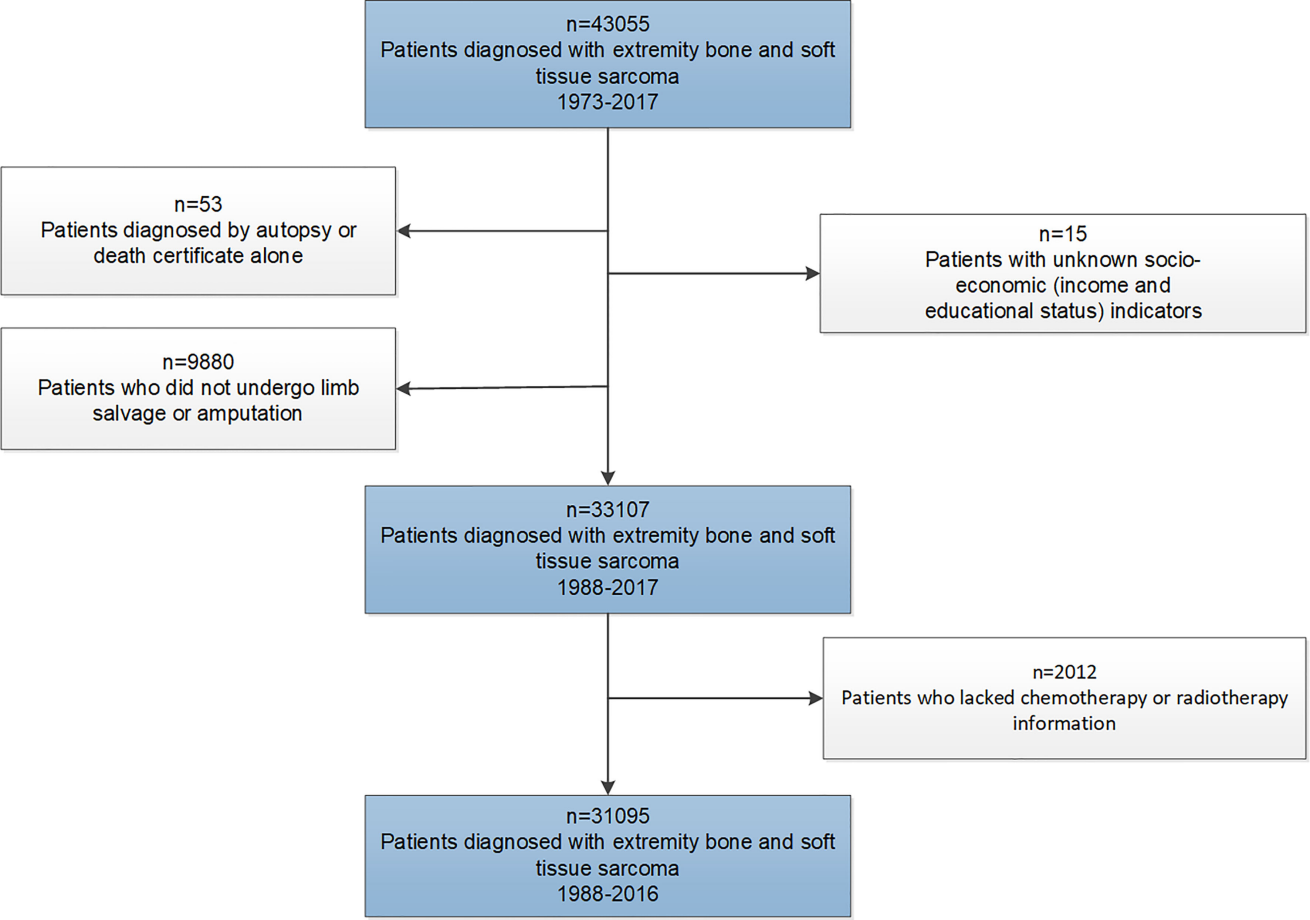
Flowchart describing initial dataset and exclusions leading to final study cohorts.

The SEER program was established by the National Cancer Institute for the evaluation of population-based cancer statistics in the United States. The database comprised 18 geographic registries, covering approximately 28% of the US population (19). This retrospective study cohort initially consisted of patients diagnosed with EBST sarcomas between January 1, 1973, and December 31, 2017, from the SEER database. Through the exclusions depicted in **Figure 1**, we identified the final study cohort. Patients diagnosed with EBST sarcomas before 1988 were also excluded, as these cases lacked treatment information for surgery.

We extracted the data of demographic characteristics including age at diagnosis, sex (female and male), race (white, black, and other), calendar year of diagnosis (1988–1999, 2000–2009, and 2010–2017), marital status at diagnosis (married, unmarried, and unknown), insurance status (insured, any Medicaid, uninsured, and unknown), and socioeconomic indicators (income and educational status). Income (median family income) and educational level (percentage of residents >25 years of age with at least a high school degree) from county-level data were calculated by linking to the 2000 United States Census and categorized into tertiles (20). The tumor-related characteristics included laterality (left, right, and others), primary site (upper limb and lower limb), grade (Grade I–IV), clinical stage of sarcoma (local, regional, distant, and unknown), and information regarding treatment. All sarcomas were classified into ten histological subtypes according to the International Classification of Disease for Oncology third revision (ICD-O-3) (21, 22), including chondrosarcoma, osteosarcoma, Ewing sarcoma, liposarcoma, malignant fibro histiocytoma (MFH), leiomyosarcoma, fibrosarcoma, synovial sarcoma, and Malignant Peripheral Nerve Sheath Tumor (MPNST) and others (ICD-O-3 codes are listed in **Table 1**). Survival time and cause of death were also available.

**Table 1:** Patient information based on their baseline characteristics before and after 1:1 PSM among subgroups receiving limb salvage and amputation.

Characteristic	Before PSM				After PSM			
	Total No.	Amputation No. (%)	Limb Salvage No. (%)	P value	Total No.	Amputation No. (%)	Limb Salvage No. (%)	P value
**All patients**	31095	3327 (100)	27768 (100)	**-**	6654	3327 (100)	3327 (100)	
**Age (years)**								
Median	53	44	54	<0.001	44	44	44	0.873
**Year**				<0.001				0.837
1988-1999	5740	819 (25)	4921 (18)		1622	819 (25)	803 (24)	
2000-2009	14042	1511 (45)	12531 (45)		3045	1511 (45)	1534 (46)	
2010-2017	11313	997 (30)	10316 (37)		1987	997 (30)	990 (30)	
**Sex**				<0.001				0.635
Female	14138	1334 (40)	12804 (46)		2687	1334 (40)	1353 (41)	
Male	16957	1993 (60)	14964 (54)		3967	1993 (60)	1974 (59)	
**Race** **^a^**				<0.001				0.340
White	25011	2630 (79)	22381 (81)		5304	2630 (79)	2674 (80)	
Black	3407	433 (13)	2974 (10)		849	433 (13)	416 (13)	
Others	2677	264 (8)	2413 (9)		501	264 (8)	237 (7)	
**Marital status**				<0.001				0.473
Married	15146	1274 (38)	13872 (50)		2504	1274 (38)	1230 (37)	
Unmarried	14568	1939 (58)	12629 (46)		3927	1939 (58)	1988 (60)	
Unknown	1381	114 (3)	1267 (5)		223	114 (3)	109 (3)	
**Education** **^b^**				<0.001				0.881
High	10736	1246 (38)	9490 (34)		2497	1246 (38)	1251 (38)	
Median	10318	1109 (33)	9209 (33)		2231	1109 (33)	1122(33)	
Low	10041	972 (29)	9069 (33)		1926	972 (29)	954 (29)	
**Income** **^b^**				<0.001				0.351
High	9965	973 (29)	8992 (32)		1894	973 (29)	921 (28)	
Median	9191	985 (30)	8206 (30)		1981	985 (30)	996 (30)	
Low	11939	1369 (41)	10570 (38)		2779	1369 (41)	1410 (42)	
**Insurance**				<0.001				0.993
Insured	10468	822 (25)	9646 (35)		1639	822 (25)	817 (25)	
Any Med	2308	362 (11)	1946 (7)		729	362 (11)	367 (11)	
Uninsured	501	53 (2)	448 (2)		108	53 (2)	55 (2)	
Unknown	17818	2090 (63)	15728 (57)		4178	2090 (63)	2088 (63)	
**Location** **^c^**				<0.001				0.523
Upper Limb	8554	762 (23)	7792 (28)		1546	762 (23)	784 (24)	
Lower Limb	22541	2565 (77)	19976 (72)		5108	2565 (77)	2543 (76)	
**Laterality**				0.699				0.189
Left	15715	1674 (50)	14041 (51)		3326	1674 (50)	1652 (50)	
Right	15337	1650 (50)	13687 (49)		3325	1650 (50)	1675 (50)	
Unknown	43	3 (0)	40 (0)		3	3 (0)	0 (0)	
**Grade** **^d^**				<0.001				0.843
Grade I	4555	173 (5)	4382 (16)		336	173 (5)	163 (5)	
Grade II	4821	374 (11)	4447 (16)		770	374 (11)	396 (12)	
Grade III	5185	687 (21)	4498 (16)		1389	687 (21)	702 (21)	
Grade IV	8369	1172 (35)	7197 (26)		2341	1172 (35)	1169 (35)	
Unknown	8165	921 (28)	7244 (26)		1818	921 (28)	897 (27)	
**Stage**				<0.001				0.909
Localized	20599	1273 (38)	19326 (70)		2555	1273 (38)	1282 (38)	
Regional	7005	1370 (41)	5635 (20)		2721	1370 (41)	1351 (41)	
Distant	2178	499 (15)	1679 (6)		996	499 (15)	497 (15)	
Unknown	1313	185 (6)	1128 (4)		382	185 (6)	197 (6)	
**Histology^e^**				<0.001				0.633
Chondrosarcoma	2431	324 (10)	2107 (8)		685	324 (10)	361 (11)	
Osteosarcoma	3534	927 (28)	2607 (9)		1877	927 (28)	950 (29)	
Ewing sarcoma	745	119 (4)	626 (2)		251	119 (4)	132 (4)	
Liposarcoma	5377	141 (4)	5236 (19)		254	141 (4)	113 (3)	
MFH	4041	316 (9)	3725 (13)		648	316 (9)	332 (10)	
Leiomyosarcoma	2619	120 (4)	2499 (9)		243	120 (4)	123 (4)	
Fibrosarcoma	2043	108 (3)	1935 (7)		221	108 (3)	113 (3)	
Synovial sarcoma	1852	332 (10)	1520 (6)		622	332 (10)	290 (9)	
MPNST	714	70 (2)	644 (2)		140	70 (2)	70 (2)	
Others	7739	870 (26)	6869 (25)		1713	870 (26)	843 (25)	
**Radiation**				<0.001				0.666
Yes	12149	451 (14)	11698 (42)		890	451 (14)	439 (13)	
No/Unknown	18946	2876 (86)	16070 (58)		5764	2876 (86)	2888 (87)	
**Chemotherapy**				<0.001				0.769
Yes	8510	1645 (49)	6865 (25)		3302	1645 (49)	1657 (50)	
No/Unknown	22585	1682 (51)	20903 (75)		3352	1682 (51)	1670 (50)	

PSM, Propensity score-matched; MFH, malignant fibrous histiocytoma; MPNST, malignant peripheral nerve sheath tumor.

a Others included American Indian/AK Native, Asian/Pacific Islander and unknown race.

b Education status and income level were categorized into tertiles.

c Upper Limb included C40.0-C40.1, C47.1 and C49.1; Lower Limb included C40.2-C40.3, C47.2 and C49.2.

d Grade I, Well differentiated; Grade II, Moderately differentiated; Grade III, Poorly differentiated; Grade IV, Undifferentiated; anaplastic.

e ICD-O-3: Chondrosarcoma, 9220-9243; Osteosarcoma, 9180-9200; Ewing sarcoma, 9260; Liposarcoma, 8850-8858; MFH, 8830; Leiomyosarcoma, 8890-8891 and 8896; Synovial sarcoma, 9040-9044; MPNST, 9540 and 9561.

To analyze the changes in the incidence of limb salvage over the calendar years, we conducted the joinpoint regression analysis program. The joinpoint model was used to calculate Annual Percentage Change (APC), mean APC, and corresponding 95% CIs of surgery rate. Statistical significance of the APC was determined by *t*-test compared with zero. To determine the subgroups of sarcoma patients who tended to receive LSS, we also calculated odds ratios (ORs) with 95% CIs based on the logistic regression model.

We used the Student’s *t*-test for continuous variables and Pearson’s chi-square test for categorical variables to compare the differences between groups. We analyzed OS, cancer-specific survival (CSS), and non-sarcoma survival (NSS) using the Kaplan–Meier method and log-rank analysis. Cumulative incidence curves were plotted and compared using the Gray test. We constructed logistic regression models to identify factors for higher incidence of LSS among sarcomas patients.

The propensity score-matched analyses were performed to compare the outcomes of patients receiving LSS and those receiving amputation. One-to-one matching without replacement was completed using the nearest-neighbor match on the logit of the propensity score for amputation administration (derived from age, sex, race, year, marital status, socioeconomic indicators, insurance status, primary site, laterality, grade, stage, histology type, radiotherapy, and chemotherapy). The caliper width was 0.05 times the standard deviation of the logit of the propensity score, which could eliminate greater than 99% of the bias due to confounding factors (23, 24). Patient characteristics were well balanced among all covariates (**Table 1**).

To account for potential biases favoring the administration of LSS to patients with more favorable baseline prognoses, sequential landmark analyses assessing the survival of patients receiving LSS and amputation were performed for patients surviving a minimum of ≥1, ≥3, ≥5, and ≥10 years from diagnosis.

The number of deaths from non-cancer diseases divided by person-years of survival was calculated as the mortality rate of non-cancer diseases. For comparison, the mortality data of the general US population collected by the National Center for Health Statistics spanning from 1969 to 2018 were used. Standardized mortality rates (SMRs) were calculated as the ratios of the observed to the expected number of deaths, which provided the relative risk of death from non-sarcoma diseases for cancer patients compared with the general US population after adjusting the basic confounding factors including age, sex, and race (25, 26). A 5-year age range was used for standardization, and the 95% CI of SMR was determined using the Poisson distribution approximation.

Observations were censored if patients did not die from indicated events at the time of the last follow-up. The survival time was from diagnosis until the occurrence of all-cause death, cancer death, or censor events, and that recorded as 0 month in the SEER database was converted to one-half of a month based on accepted epidemiologic practices (27). All statistical tests were two-sided, and values with *p* < 0.05 were considered statistically significant.

The SEER database was accessed using SEER*Stat software 8.3.8. The Student’s *t*-test, Pearson’s chi-square test, propensity score matching, logistic regression analyses, Cox regression analyses, Fine-Gray model, Gray tests, subgroup analysis, and interaction test were conducted using R version 4.0.3. The Kaplan–Meier survival curves, cumulative incidence curves, and log-rank analysis were performed using GraphPad Prism 8.0.

## Results

A total of 31,095 patients diagnosed with EBST sarcomas (6,717 individuals with bone sarcomas and 24,378 individuals with soft tissue sarcomas) from 1988 to 2017 were identified in this study, followed by 2,619,290 person-years. In the study cohort, 3,327 patients (10.7%) underwent amputation for EBST sarcomas with a median follow-up time of 42 months (interquartile range [IQR], 15–117 months), and 27,768 (89.3%) patients underwent LSS for sarcomas with a median follow-up time of 62 months (IQR, 23–131 months).

### The Incidence of Limb Salvage With Calendar Year Among Patients With EBST Sarcomas

**Figure 2:**
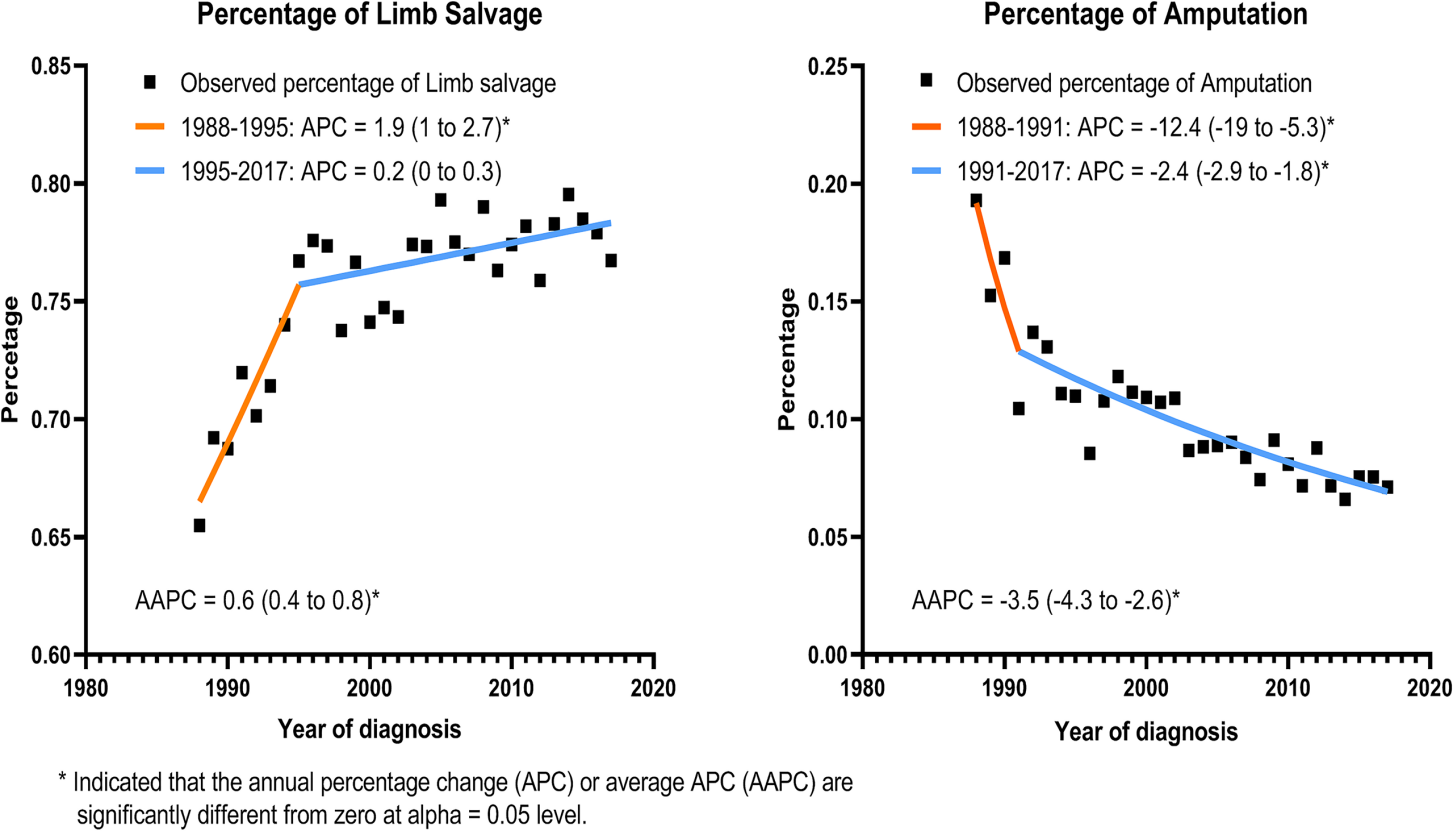
Incidence of limb salvage and amputation with calendar year among all patients with EBST sarcomas.

Using the joinpoint model, **Figure 2** demonstrated that the percentage of LSS procedures increased significantly from 65.5% in 1988 to 76.7% in 1995 (APC, 1.9%; 95% CI, 1 to 2.7; *p* < 0.05), and then showed a nonsignificant increase from 1995 to 2017 (APC, 0.2%; 95% CI, 0 to 0.3; *p* < 0.06). The proportion of amputation decreased remarkably from 19.3% in 1988 to 10.5% in 1991 (APC, −12.4%; 95% CI, −19 to −5.3; *p* < 0.05), and then slowly returned from 1991 to 7.1% in 2017 (APC, −2.4%; 95% CI, −2.9 to −1.8; *p* < 0.05).

### The Subgroups of Sarcoma Patients Tending to Receive Limb Salvage Procedures

**Table 2** shows the ORs of patients who were more likely to receive limb salvage, stratified by subgroups. The younger patients had a higher OR for the tendency to receive LSS compared to older patients (OR = 0.997; 95% CI [0.995–0.999]; *p* = 0.007). Patients with upper limb sarcoma had an OR of 1.254 (95% CI [1.147–1.373]) compared to those with lower limb sarcoma. Compared with those with chondrosarcoma, patients with fibrosarcoma (OR = 2.529; 95% CI [2.011–3.205]; *p* < 0.001), leiomyosarcoma (OR = 3.352; 95% CI [2.683–4.212]; *p* < 0.001), liposarcoma (OR = 4.999; 95% CI [4.060–6.184]; *p* < 0.001), malignant fibro-histiocytoma (OR = 2.535; 95% CI [2.120–3.032]; *p* < 0.001), and MPNST (OR = 1.624; 95% CI [1.229–2.172]; *p* = 0.001) were more prone to receive LSS after sarcoma diagnosis.

**Table 2:** Odds ratios of limb salvage procedures compared with amputation among patients with sarcomas. .

	Logistic regression model		
	**Odds ratio**	**95%CI**	**P value**
**Age at diagnosis**	0.997	(0.995, 0.999)	0.007
**Sex**			
Male	–		
Female	1.269	(1.174, 1.371)	<0.001
**Race**			
White	–		
Black	0.957	(0.852, 1.078)	0.468
Other	1.124	(0.977, 1.298)	0.105
**Year of diagnosis**			
1988-1999	–		
2000-2009	1.546	(1.395, 1.713)	<0.001
2010-2017	1.741	(1.511, 2.005)	<0.001
**Marital status**			
Unmarried	–		
Married	1.182	(1.083, 1.290)	<0.001
Unknow	1.024	(0.833, 1.269)	0.824
**Education**			
High	–		
Median	1.103	(0.994, 1.223)	0.065
Low	1.251	(1.113, 1.406)	<0.001
**Income**			
High	–		
Median	0.912	(0.826, 1.008)	0.070
Low	0.979	(0.876, 1.093)	0.706
**Insurance**			
Any Medicaid	–		
Insured	1.506	(1.303, 1.737)	<0.001
Uninsured	1.272	(0.930, 1.769)	0.141
Unknown	1.219	(1.047, 1.418)	0.010
**Location**			
Lower Limb	–		
Upper Limb	1.254	(1.147, 1.373)	<0.001
**Laterality**			
Left	–		
Right	0.985	(0.913, 1.062)	0.695
Others	2.565	(0.895, 10.831)	0.125
**Grade**			
Grade IV	–		
Grade I	1.918	(1.597, 2.313)	<0.001
Grade II	1.325	(1.157, 1.520)	<0.001
Grade III	0.951	(0.855, 1.059)	0.363
Unknown	1.152	(1.040, 1.276)	0.007
**Stage**			
Distant	–		
Localized	2.854	2.525, 3.221	<0.001
Regional	1.035	0.916, 1.167	0.579
Unknow	1.553	1.283, 1.887	<0.001
**Histology**			
Chondrosarcoma	–		
Osteosarcoma	0.726	0.614, 0.858	<0.001
Ewing sarcoma	1.208	0.940, 1.559	0.144
Liposarcoma	4.999	4.060, 6.184	<0.001
MFH	2.535	2.120, 3.032	<0.001
Leiomyosarcoma	3.352	2.683, 4.212	<0.001
Fibrosarcoma	2.529	2.011, 3.205	<0.001
Synovial sarcoma	0.809	0.674, 0.971	0.023
MPNST	1.624	1.229, 2.172	0.001
Others	1.390	1.194, 1.614	<0.001

Abbreviations: MFH, malignant fibrous histiocytoma; MPNST, malignant peripheral nerve sheath tumor.

### Comparison of Survival Among Patients After Limb Salvage and Amputation Due to EBST Sarcomas

**Figure 3:**
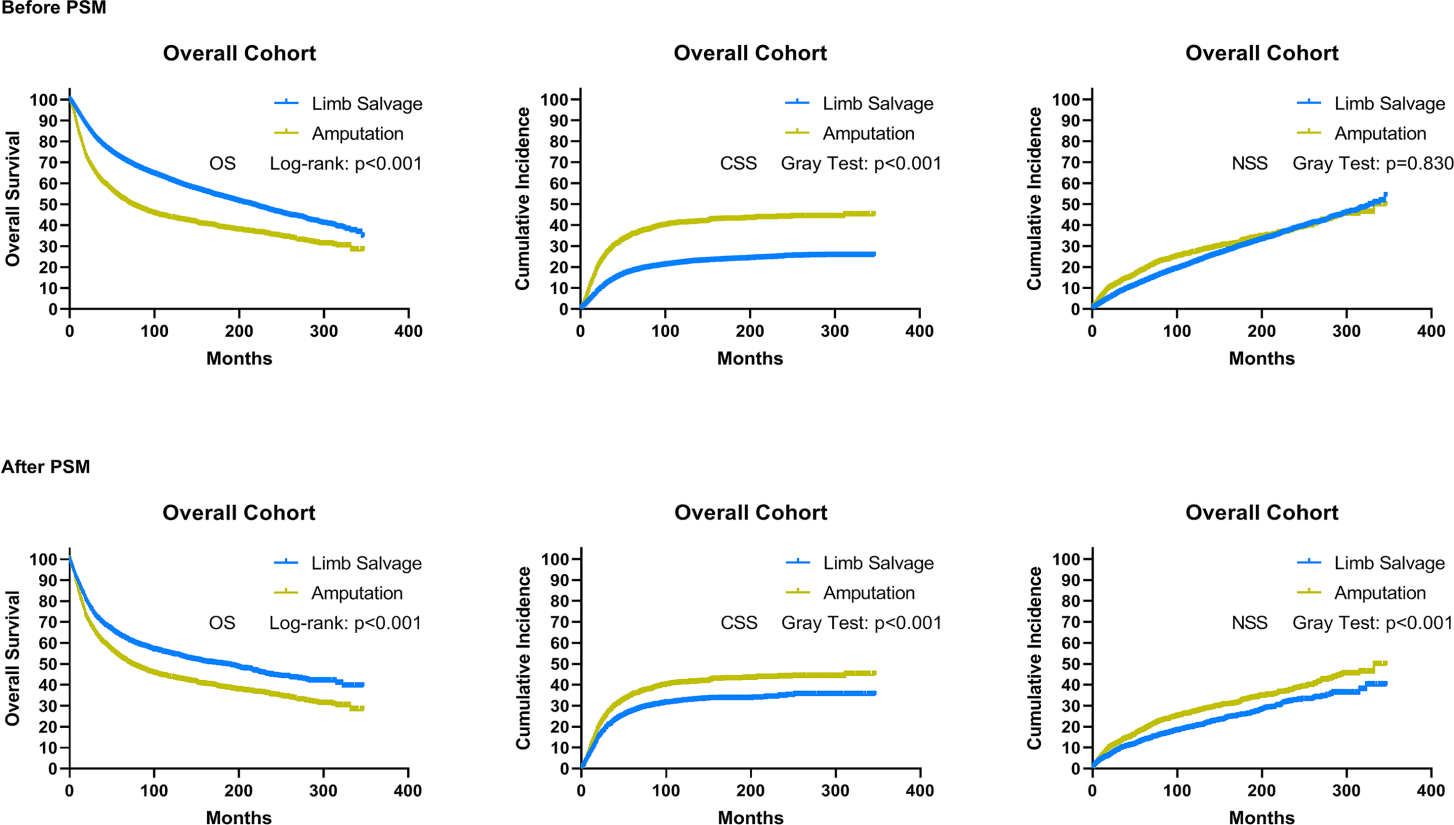
As a whole, overall survival, cancer-specific survival, and non-sarcoma survival for patients receiving limb salvage or amputation after EBST sarcomas using the initial cohort and cohort after PSM, respectively. PSM, propensity score-matched patients; OS, overall survival; CSS, cancer-specific survival; NSS, non-sarcomas survival.

Patients who underwent LSS had improved OS and CSS compared with those who underwent amputation (**Supplementary Table 1**), and there was no statistically significant difference in NSS between the two groups (**Figure 3**). A propensity score analysis was performed to match 3,327 patients who underwent LSS with 3,327 patients who underwent amputation. This matched analysis demonstrated a significant association between LSS and improvements in overall OS (*p* < 0.001), CSS (*p* < 0.001), and NSS (*p* < 0.001) in sarcoma patients.

**Figure 4:**
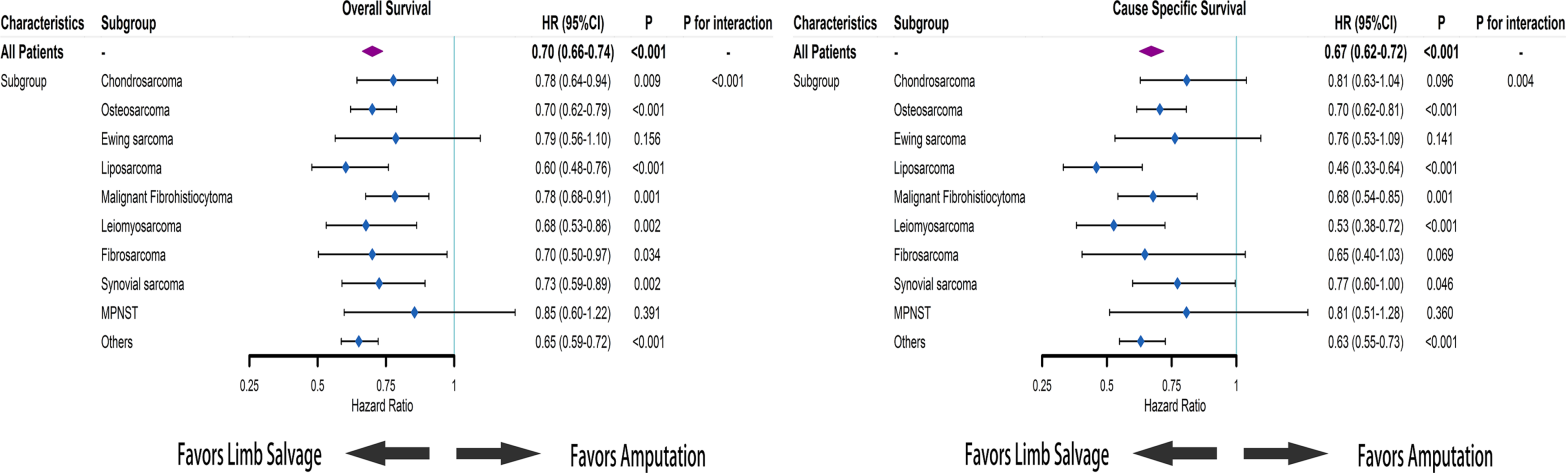
Based on Cox regression model, forest plots of the association between limb salvage after sarcomas and survival in different sarcoma subgroups. HR, hazard ratio; CI, confidence interval.

Using the initial cohort, we conducted the subgroup analysis based on subtypes of sarcomas. Survival curves and cumulative incidence curves are drawn in **Supplementary Figure 1**. In the subgroup analysis, the OS of receiving limb salvage did not differ significantly from the OS of receiving amputation in patients with Ewing sarcoma and MPNST (HR = 0.79; 95% CI [0.56–1.10]; *p* = 0.156 and HR = 0.85; 95% CI [0.60–1.22]; *p* = 0.391). For patients with other sarcoma subtypes, limb salvage was associated with improved OS (**Figure 4**). The limb salvage was also associated with better CSS than amputation among patients with osteosarcoma (HR = 0.70; 95% CI [0.62–0.81]; *p* < 0.001), liposarcoma (HR = 0.46; 95% CI [0.33–0.64]; *p* < 0.001), MFH (HR = 0.68; 95% CI [0.54–0.85]; *p* = 0.001), and leiomyosarcoma (HR = 0.53; 95% CI [0.38–0.72]; *p* < 0.001). Among patients with chondrosarcoma (HR = 0.81; 95% CI [0.63–1.04]; *p* = 0.096), Ewing sarcoma (HR = 0.76; 95% CI [0.53–1.09]; *p* = 0.141), fibrosarcoma (HR = 0.65; 95% CI [0.40–1.03]; *p* = 0.069), synovial sarcoma (HR = 0.77; 95% CI [0.60–1.00]; *p* = 0.046), and MPNST (HR = 0.81; 95% CI [0.51–1.28]; *p* = 0.360), no CSS benefit could be demonstrated after limb salvage compared with amputation. Using propensity score-matched cohorts, we conducted the subgroup analysis based on the subtype and metastatic status of sarcomas. For sarcoma patients without distant metastases, LSS did not increase OS of patients with MFH, fibrosarcoma, and MPNST but was associated with better OS among patients with other sarcoma subtypes (**Supplementary Figure 2**). Furthermore, LSS increased CSS among patients with osteosarcoma (*p* = 0.023), Ewing sarcoma (*p* = 0.035), and leiomyosarcoma (*p* < 0.001) but did not improve CSS of patients with chondrosarcoma (*p* = 0.176), liposarcoma (*p* = 0.050), MFH (*p* = 0.144), fibrosarcoma (*p* = 0.281), synovial sarcoma (*p* = 0.082), and MPNST (*p* = 0.646). For patients with distant metastases, LSS only improved the survival among osteosarcoma patients (OS: *p* < 0.001 and CSS: *p* = 0.001) but worsened the survival of patients with MPNST (OS: *p* = 0.011 and CSS: *p* = 0.049). In addition, there was no apparent improvement in survival in the group of patients with other sarcoma subtypes who received LSS compared with those who received amputation (**Supplementary Figure 3**).

**Figure 5:**
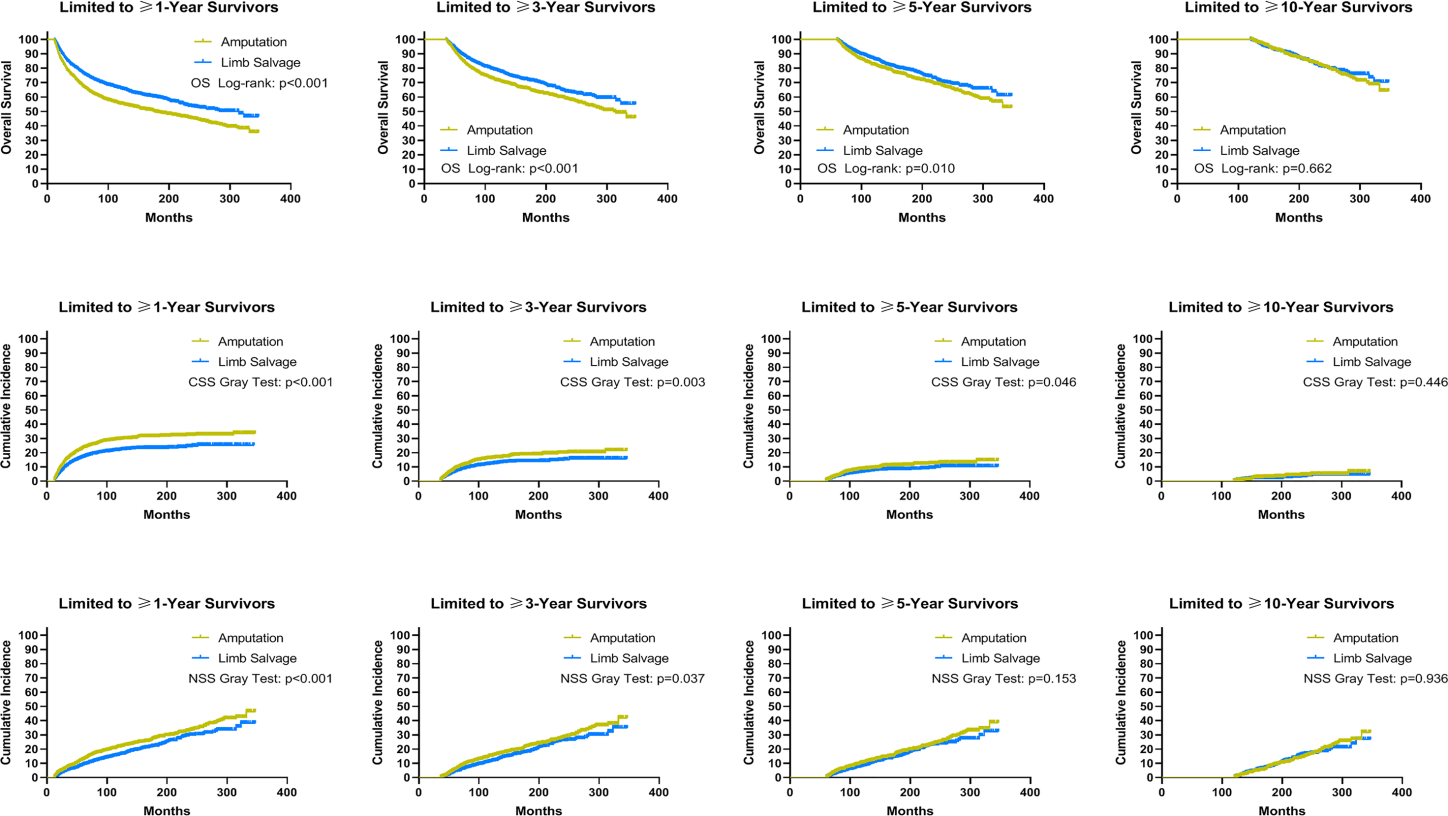
Subgroup analysis: Landmark analyses of overall survival, cancer-specific survival, and non-sarcoma survival for long-term survivors with localized and regional sarcomas in the cohort after PSM. PSM, propensity score-matched patients; OS, overall survival; CSS, cancer-specific survival; NSS, non-sarcoma survival.

**Figure 6:**
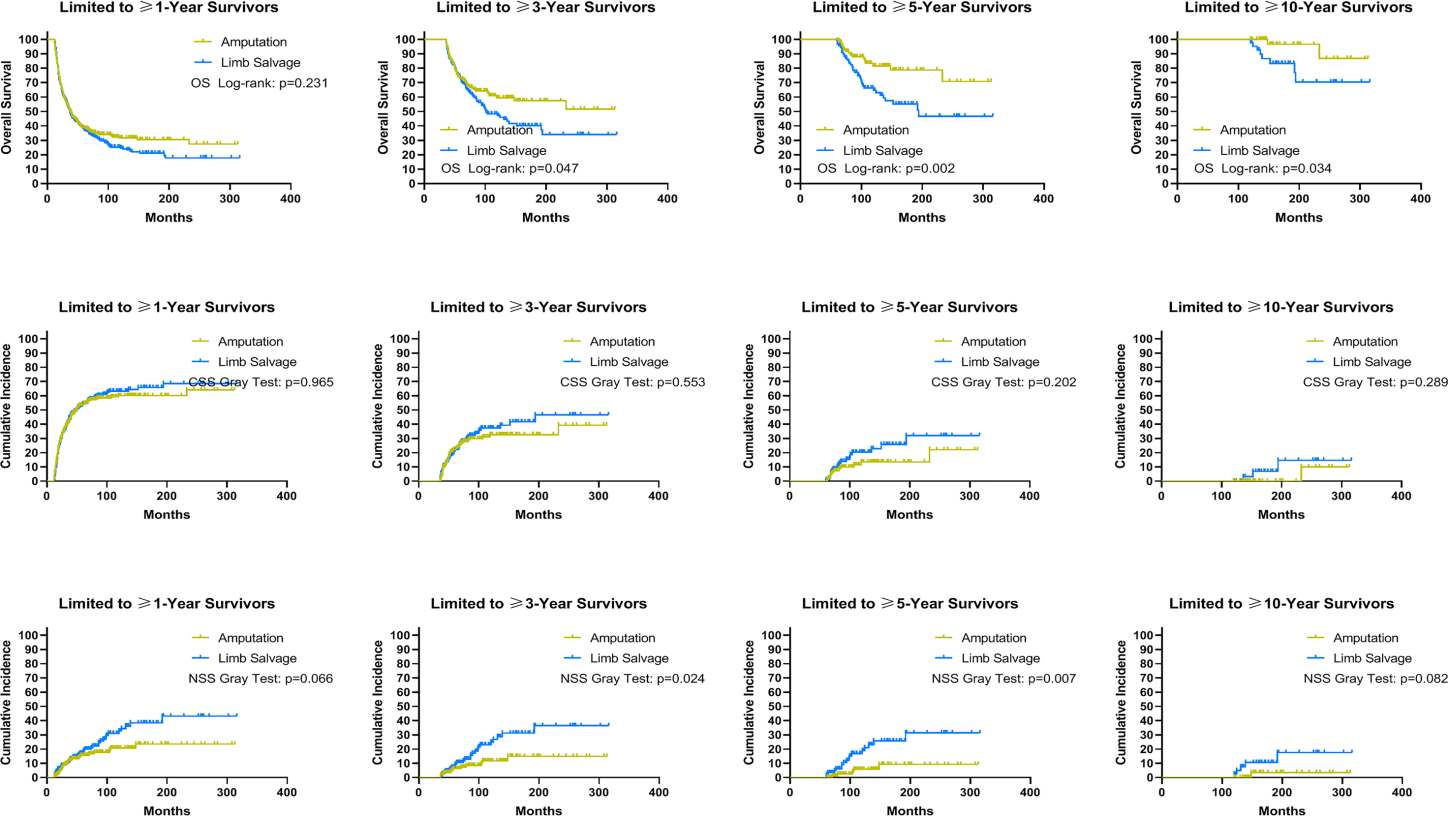
Subgroup analysis: Landmark analyses of overall survival, cancer-specific survival, and non-sarcoma survival for long-term survivors with advanced sarcomas in the cohort after PSM. PSM, propensity score-matched patients; OS, overall survival; CSS, cancer-specific survival; NSS, non-sarcoma survival.

Using data from the PSM cohort after adjusting for other confounding factors, we found that for patients without distant metastases, LSS was associated with improved OS and CSS for survivors at ≥1, ≥3, and ≥5 years, as well as NSS for survivors at ≥1 and ≥3 years since diagnosis, compared to amputation (**Figure 5**). For patients with distant metastases, in comparison with amputation, LSS was associated with worse OS for survivors of ≥3, ≥5, and ≥10 years and NSS for survivors of ≥3 and ≥5 years, while having no effect on CSS for survivors of ≥1, ≥3, ≥5, and ≥10 years (**Figure 6**). For osteosarcoma patients with distant metastases, LSS did not improve CSS for survivors of ≥1, ≥3, ≥5, and ≥10 years (P=0.249, P=0.546, P=0.893, P>0.900).

Since amputation was associated with a worse prognosis among sarcoma patients with distant metastases, we also analyzed non-sarcoma death. A total of 5,644 patients with EBST sarcomas died from non-sarcoma diseases. Of these, cardiovascular diseases had the highest mortality rate, followed by infectious diseases and chronic obstructive pulmonary disease (COPD) (**Supplementary Table 2**). Compared with the general US population, patients with EBST sarcomas had a higher risk of dying from these non-cancer causes except for renal diseases (**Supplementary Table 3**). Patients who had received LSS had a higher risk of dying from cardiovascular diseases, COPD, diabetes mellitus, and Alzheimer’s disease, and those who had an amputation had a higher risk of dying from accident injuries, infectious diseases, and suicide (**Table 3**).

**Table 3:** The comparison of the risk of dying from some non-cancer diseases between survivors after limb salvage and those after amputation.

	CVD	COPD	Accidental death	Infection	DM	Suicide	AD	ND
**Amputation**								
Number of deaths	132	16	18	29	8	10	7	5
Mortality	626.20	75.90	85.39	137.57	37.95	47.44	33.21	23.72
SMR	1.97	1.67	2.13	5.87	1.53	3.61	2.08	1.82
95% CI	1.66 to 2.34	1.02 to 2.72	1.34 to 3.38	4.08 to 8.45	0.76 to 3.06	1.94 to 6.71	0.99 to 4.37	0.76 to 4.38
**Limb Salvage**								
Number of deaths	1313	185	124	191	100	40	113	48
Mortality	665.84	93.82	62.88	96.86	50.71	20.28	57.30	24.34
SMR	1.39	1.29	1.35	3.00	1.33	1.41	1.96	1.14
95% CI	1.32 to 1.47	1.12 to 1.49	1.13 to 1.60	2.61 to 3.46	1.09 to 1.62	1.03 to 1.92	1.63 to 2.35	0.86 to 1.51

CVD, cardiovascular diseases; COPD, chronic obstructive pulmonary diseases; DM, diabetes mellitus; AD, Alzheimer’s disease; KD, kidney disease; SMR, standardized mortality ratio; CI, confidence interval.

## Discussion

The treatment of sarcoma patients is evolving with the development of multimodality therapy, and advances in imaging techniques and the use of neoadjuvant chemotherapy have resulted in a greater frequency of limb salvage procedures (12, 28), which is consistent with our results in this study. Several small samples or single-center clinical cohort studies have shown that LSS had a better prognosis than amputation among patients with EBST sarcomas (13, 29). In addition to the postoperative survival rate, clinicians are increasingly concerned about the higher recurrence rate and complications caused by limb salvage procedures, as these are associated with a poorer prognosis (9, 10, 30). Some studies have reported no significant difference in the incidence of local recurrence of sarcoma among patients who underwent LSS and amputation (8, 29). However, several other studies have implicated LSS with a higher incidence of local recurrence and overall complications (31, 32). Therefore, there is much debate about the impact of LSS on the survival of EBST sarcomas. Moreover, due to the limitations of the small sample or single center of these studies, no results were available to inform clinical practice currently. To our knowledge, this analysis represents the largest reported cohort of patients with bone and soft tissue sarcoma treated with LSS or amputation. Using data from the SEER database, we found that the impact of LSS on the prognosis of patients with EBST sarcoma differed markedly depending on the subtypes and clinical stage of sarcoma. Our comparative study was inconsistent with the traditional views of surgical radicality, in which amputation and safe resection margin were critical for better prognosis and lower local recurrence (33, 34). Considering the heterogeneity among EBST sarcomas, we performed the subgroup analysis. We found that LSS could improve the survival of patients with osteosarcoma, regardless of whether they have distant metastases. However, in general, patients with advanced sarcomas are at an increased risk of dying from a range of non-sarcoma diseases after LSS, such as cardiovascular diseases, COPD, diabetes mellitus, and Alzheimer’s disease. For complete removal of sarcoma, surgeons always recommend amputation to patients with advanced osteosarcoma, equivalent to clinical stage III (6). The results of this study highlighted a new concept that surgeons should consider actively LSS for patients with osteosarcoma at any stage for better prognosis.

For soft tissue sarcomas, subgroup analysis showed that LSS increased the OS rate among patients with localized and regional liposarcomas, leiomyosarcomas, and synovial sarcomas compared with amputation, but no survival difference was observed among patients with advanced soft tissue sarcomas other than MPNST. LSS had a deleterious effect on the survival of those with advanced MPNST, one of the most challenging mesenchymal malignancies to treat and predisposed to early metastasis. In the early stage of disease, the relapse rates were high following multimodality therapy. In advanced diseases, the response rates to cytotoxic chemotherapy were low. In our study, the poor survival of MPNST patients receiving LSS could be attributed to the agressivity of the disease, high recurrence, and lack of chemosensibility due to mutation of the NF1 gene (35, 36). Moreover, the landmark analysis for survivors with advanced osteosarcomas of ≥1 year revealed no effect of LSS on survival, which indicated that patients could benefit from LSS only within 1 year after sarcoma diagnosis.

Using data from SEER database, we also found that the patients receiving amputation after sarcomas had a higher risk of suicide and accidental death than those receiving LSS. This may be explained by the higher degree of depression and demoralization caused by the altered gait, function, stability, strength, and appearance resulting from amputation (37–39). Therefore, clinicians should take some measures including long-lasting follow up and psychological support to lower the risk of death from non-sarcoma diseases such as suicide (39, 40).

There are some limitations in our study. First, the SEER database does not contain detailed quantitative data on Patient Reported Outcomes Measurement Information System (PROMIS), including the acceptance of postoperative state, ambulation, and pain levels, a new patient-reported scoring system developed by the National Institutes of Health that is being widely adopted. Considering that physicians’ and patients’ definitions of surgical success can vary widely, it is necessary to explore the relationship between patient-reported outcomes and LSS. Using a cohort of 43 sarcoma patients, Yannick et al. (10) reported comparable mental wellbeing between patients with LSS and those receiving amputation, although the functional benefits of LSS over amputation were maintained at nearly 10 years of follow-up. However, we could not evaluate the impact of LSS on the quality of life in these patients in detail using data from the SEER program. The relationship between patient-reported outcomes and LSS requires further study using the population-based study cohort. Second, in the subgroup analysis, we did not find any effect of LSS on NSS among patients with advanced sarcomas, but the results of landmark analysis were inconsistent with the subgroup analysis. A possible hypothesis to explain this inconsistency was that the relatively small number of non-sarcoma events reduced the statistical power of the subgroup analysis and, therefore, the effect of LSS on the risk of dying from non-sarcoma diseases requires further investigation. Moreover, the SEER program did not provide sarcoma-specific data, such as surgery margins and sarcoma depth. Nevertheless, the present study is the first large retrospective study to investigate the relationship between the use of LSS and survival of sarcoma patients in a modern cohort, and we believe that the results are reliable and can be utilized to guide clinical practice (41).

## Conclusion

Different from current clinical practice guidelines, osteosarcoma patients could benefit from limb salvage procedures regardless of whether the sarcoma has metastasized. Furthermore, clinicians should pay more attention to the high risk of dying from different non-sarcoma diseases among patients after amputation and those after LSS during the postoperative follow-up.

## Data Availability Statement

Publicly available datasets were analyzed in this study. These data can be found here: https://seer.cancer.gov/.

## Author Contributions

Conception and design of study: KY and FL. Acquisition of data: KY, YC, HG and FX. Analysis and/or interpretation of data: KY, FX, YC, KS, and YT. Drafting the manuscript: KY, YC, and FX. All authors contributed to the article and approved the submitted version.

## Conflict of Interest

The authors declare that the research was conducted in the absence of any commercial or financial relationships that could be construed as a potential conflict of interest.

## Publisher’s Note

All claims expressed in this article are solely those of the authors and do not necessarily represent those of their affiliated organizations, or those of the publisher, the editors and the reviewers. Any product that may be evaluated in this article, or claim that may be made by its manufacturer, is not guaranteed or endorsed by the publisher.

## Supplementary Material

Supplementary Figure 1 Subgroup analysis: the graph showed Kaplan-Meier curves of overall survival, and cumulative incidence curves of death resulting from sarcomas and death resulting from other non-sarcomas causes. MFH, malignant fibro histiocytoma; MPNST, malignant peripheral nerve sheath tumors.

Supplementary Figure 2 Using subgroup with localized and regional sarcomas in the cohort after PSM, the graph showed Kaplan-Meier curves of overall survival, and cumulative incidence curves of death resulting from sarcomas and death resulting from other non-sarcomas causes. MFH, malignant fibro histiocytoma; MPNST, malignant peripheral nerve sheath tumors.

Supplementary Figure 3 Using subgroup with distant metastases in the cohort after PSM, the graph showed Kaplan-Meier curves of overall survival, and cumulative incidence curves of death resulting from sarcomas and death resulting from other non-sarcomas causes. MFH, malignant fibro histiocytoma; MPNST, malignant peripheral nerve sheath tumors.

## References

[1] Kask G Barner-Rasmussen I. ASO Author Reflection: Demographic Conditions are the Major Determinants for Functional Outcome and Quality of Life in Lower Extremity Soft Tissue Sarcoma Patients. Ann Surg Oncol (2021) 28(11):6906–7. doi: 10.1245/s10434-021-09781-7 PMC8460492 33651213

[2] Casali PG Le Cesne A Velasco AP Kotasek D Rutkowski P Hohenberger P. Final Analysis of the Randomized Trial on Imatinib as an Adjuvant in Localized Gastrointestinal Stromal Tumors (GIST) From the EORTC Soft Tissue and Bone Sarcoma Group (STBSG), the Australasian Gastro-Intestinal Trials Group (AGITG), UNICANCER, French Sarcoma Group (FSG), Italian Sarcoma Group (ISG), Spanish Group for Research on Sarcomas (GEIS). Ann Oncol (2021) 32(4):533–41. doi: 10.1016/j.annonc.2021.01.004 33482247

[3] Surveillance Epidemiology and End Results (SEER) Program. Available at: https://seer.cancer.gov/canques/mortality.html.

[4] Link MP Goorin AM Miser AW Green AA Pratt CB Belasco JB. The Effect of Adjuvant Chemotherapy on Relapse-Free Survival in Patients With Osteosarcoma of the Extremity. N Engl J Med (1986) 314(25):1600–6. doi: 10.1056/nejm198606193142502 3520317

[5] Rosenberg SA Tepper J Glatstein E Costa J Baker A Brennan M. The Treatment of Soft-Tissue Sarcomas of the Extremities: Prospective Randomized Evaluations of (1) Limb-Sparing Surgery Plus Radiation Therapy Compared With Amputation and (2) the Role of Adjuvant Chemotherapy. Ann Surg (1982) 196(3):305–15. doi: 10.1097/00000658-198209000-00009 PMC1352604 7114936

[6] Quinn RH. Metastatic Bone Disease. Available at: https://orthoinfo.aaos.org/en/diseases--conditions/metastatic-bone-disease/

[7] Fiore M Ford S Callegaro D Sangalli C Colombo C Radaelli S. Adequate Local Control in High-Risk Soft Tissue Sarcoma of the Extremity Treated With Surgery Alone at a Reference Centre: Should Radiotherapy Still be a Standard? Ann Surg Oncol (2018) 25(6):1536–43. doi: 10.1245/s10434-018-6393-x 29470819

[8] Tirotta F Sayyed R Jones RL Hayes AJ. Risk Factors for the Development of Local Recurrence in Extremity Soft-Tissue Sarcoma. Expert Rev Anticancer Ther (2021) 22(1):83–95. doi: 10.1080/14737140.2022.2011723 34822313

[9] Smolle MA Leithner A Kapper M Demmer G Trost C Bergovec M. Complications, Mobility, and Quality of Life in Ankle Sarcoma Patients. Bone Joint J (2021) 103-b(3):553–61. doi: 10.1302/0301-620x.103b3.Bjj-2020-1308.R1 33641415

[10] Hoftiezer YAJ Lans J van derHeijden B Chen NC Eberlin KR Lozano-Calderón SA. Long-Term Patient-Reported Outcome Measures Following Limb Salvage With Complex Reconstruction or Amputation in the Treatment of Upper Extremity Sarcoma. J Surg Oncol (2021) 123(5):1328–35. doi: 10.1002/jso.26426 33650694

[11] Reddy KI Wafa H Gaston CL Grimer RJ Abudu AT Jeys LM. Does Amputation Offer Any Survival Benefit Over Limb Salvage in Osteosarcoma Patients With Poor Chemonecrosis and Close Margins? Bone Joint J (2015) 97-b(1):115–20. doi: 10.1302/0301-620x.97b1.33924 25568424

[12] Nagarajan R Neglia JP Clohisy DR Robison LL. Limb Salvage and Amputation in Survivors of Pediatric Lower-Extremity Bone Tumors: What are the Long-Term Implications? J Clin Oncol (2002) 20:4493–501. doi: 10.1200/jco.2002.09.006 12431974

[13] Kirilova M Klein A Lindner LH Nachbichler S Knösel T Birkenmaier C. Amputation for Extremity Sarcoma: Indications and Outcomes. Cancers (Basel) (2021) 13(20). doi: 10.3390/cancers13205125 PMC8533806 34680274

[14] Evans DR Lazarides AL Visgauss JD Somarelli JA Blazer DG 3rd Brigman BE. Limb Salvage Versus Amputation in Patients With Osteosarcoma of the Extremities: An Update in the Modern Era Using the National Cancer Database. BMC Cancer (2020) 20(1):995. doi: 10.1186/s12885-020-07502-z 33054722 PMC7557006

[15] Mavrogenis AF Abati CN Romagnoli C Ruggieri P. Similar Survival But Better Function for Patients After Limb Salvage Versus Amputation for Distal Tibia Osteosarcoma. Clin Orthop Relat Res (2012) 470:1735–48. doi: 10.1007/s11999-011-2238-7 PMC3348295 22270466

[16] Alamanda VK Crosby SN Archer KR Song Y Schwartz HS Holt GE. Amputation for Extremity Soft Tissue Sarcoma Does Not Increase Overall Survival: A Retrospective Cohort Study. Eur J Surg Oncol (2012) 38(12):1178–83. doi: 10.1016/j.ejso.2012.08.024 22985713

[17] Bacci G Ferrari S Lari S Mercuri M Donati D Longhi A. Osteosarcoma of the Limb. Amputation or Limb Salvage in Patients Treated by Neoadjuvant Chemotherapy. J Bone Joint Surg Br (2002) 84(1):88–92. doi: 10.1302/0301-620x.84b1.12211 11837839

[18] Tsuda Y Lowe M Evans S Parry MC Stevenson JD Fujiwara T. Surgical Outcomes and Prognostic Factors of non-Metastatic Radiation-Induced Sarcoma of Bone. Eur J Surg Oncol (2020) 46(2):293–8. doi: 10.1016/j.ejso.2019.10.036 31703834

[19] Surveillance Epidemiology and End Results (SEER) Program: Overview of the SEER Program . Available at: http://www.seer.cancer.gov.

[20] Bureau, U. S. C. Census 2000 Gateway. Available at: http://www.census.gov/main/www/cen2000html

[21] Jacobs AJ Michels R Stein J Levin AS. Improvement in Overall Survival From Extremity Soft Tissue Sarcoma Over Twenty Years. Sarcoma (2015) 2015:279601. doi: 10.1155/2015/279601 25821397 PMC4363656

[22] Smith GM Johnson GD Grimer RJ Wilson S. Trends in Presentation of Bone and Soft Tissue Sarcomas Over 25 Years: Little Evidence of Earlier Diagnosis. Ann R Coll Surg Engl (2011) 93:542–7. doi: 10.1308/147870811x13137608455055 PMC3604925 22004638

[23] Austin PC. Optimal Caliper Widths for Propensity-Score Matching When Estimating Differences in Means and Differences in Proportions in Observational Studies. Pharm Stat (2011) 10:150–61. doi: 10.1002/pst.433 PMC3120982 20925139

[24] Cochran WG Rubin DB. Controlling Bias in Observational Studies: A Review. Sankhya Ser A (1973) 35:417–46. doi: 10.1017/CBO9780511810725.005

[25] Misono S Weiss NS Fann JR Redman M Yueh B. Incidence of Suicide in Persons With Cancer. J Clin Oncol (2008) 26:4731–8. doi: 10.1200/JCO.2007.13.8941 PMC2653137 18695257

[26] Yang K Zheng Y Peng J Chen J Feng H Yu K. Incidence of Death From Unintentional Injury Among Patients With Cancer in the United States. JAMA Netw Open (2020) 3(2):e1921647. doi: 10.1001/jamanetworkopen.2019.21647 32083692 PMC7043194

[27] Koepsell TD Weiss NS. Epidemiologic Methods: Studying the Occurrence of Illness New York: NY, Oxford University Press (2003).

[28] Renard AJ Veth RP Schreuder HW van Loon CJ Koops HS van Horn JR. Function and Complications After Ablative and Limb-Salvage Therapy in Lower Extremity Sarcoma of Bone. J Surg Oncol (2000) 73(4):198–205. doi: 10.1002/(sici)1096-9098(200004)73:4<198::aid-jso3>3.0.co;2-x 10797332

[29] Okajima K Kobayashi H Okuma T Arai S Zhang L Hirai T. Prognosis and Surgical Outcome of Soft Tissue Sarcoma With Malignant Fungating Wounds. Jpn J Clin Oncol (2021) 51(1):78–84. doi: 10.1093/jjco/hyaa176 33037437

[30] Papakonstantinou E Stamatopoulos A Athanasiadis DI Kenanidis E Potoupnis M Haidich AB. Limb-Salvage Surgery Offers Better Five-Year Survival Rate Than Amputation in Patients With Limb Osteosarcoma Treated With Neoadjuvant Chemotherapy. A Systematic Review and Meta-Analysis. J Bone Oncol (2020) 25:100319. doi: 10.1016/j.jbo.2020.100319 33088699 PMC7567946

[31] Han J Yu Y Wu S Wang Z Zhang W Zhao M. Clinical Factors Affecting Prognosis of Limb Osteosarcoma in China: A Multicenter Retrospective Analysis. J Int Med Res (2020) 48(8):300060520930856. doi: 10.1177/0300060520920053 PMC7469730 32865070

[32] Heer J Allison DC Helmstedter CS. Factors, Treatments, and Outcomes Associated With Primary Soft Tissue Malignancies of the Forearm: A Series of 31 Cases. J Orthop (2021) 28:58–61. doi: 10.1016/j.jor.2021.11.001 34840483 PMC8605106

[33] Kurisunkal V Laitinen MK Kaneuchi Y Kapanci B Stevenson J Parry MC. Is 2 Mm a Wide Margin in High-Grade Conventional Chondrosarcomas of the Pelvis? Bone Joint J (2021) 103-b(6):1150–4. doi: 10.1302/0301-620x.103b6.Bjj-2020-1869.R1 34058869

[34] Hagi T Nakamura T Nagano A Koike H Yamada K Aiba H. Clinical Outcome in Patients Who Underwent Amputation Due to Extremity Soft Tissue Sarcoma: Tokai Musculoskeletal Oncology Consortium Study. Jpn J Clin Oncol (2022) 52(2):157–62 doi: 10.1093/jjco/hyab184 34875695

[35] Somatilaka BN Sadek A McKay RM Le LQ. Malignant Peripheral Nerve Sheath Tumor: Models, Biology, and Translation. Oncogene (2022) 41(17):2405–21. doi: 10.1038/s41388-022-02290-1 PMC9035132 35393544

[36] Knight SWE Knight TE Santiago T Murphy AJ Abdelhafeez AH. Malignant Peripheral Nerve Sheath Tumors-A Comprehensive Review of Pathophysiology, Diagnosis, and Multidisciplinary Management. Children (Basel Switzerland) (2022) 9(1). doi: 10.3390/children9010038 PMC8774267 35053663

[37] Fauske L Bruland OS Grov EK Bondevik H. Cured of Primary Bone Cancer, But at What Cost: A Qualitative Study of Functional Impairment and Lost Opportunities. Sarcoma (2015) 2015:484196. doi: 10.1155/2015/484196 25949211 PMC4407620

[38] Fauske L Lorem G Grov EK Bondevik H. Changes in the Body Image of Bone Sarcoma Survivors Following Surgical Treatment–A Qualitative Study. J Surg Oncol (2016) 113:229–34. doi: 10.1002/jso.24138 PMC4736459 26714610

[39] Schreiber D Bell RS Wunder JS O'Sullivan B Turcotte R Masri BA. Evaluating Function and Health Related Quality of Life in Patients Treated for Extremity Soft Tissue Sarcoma. Qual Life Res (2006) 15(9):1439–46. doi: 10.1007/s11136-006-0001-4 16732468

[40] Yu K Wu B Chen Y Kang H Song K Dong Y. Suicide and Accidental Deaths Among Patients With Primary Malignant Bone Tumors. J Bone Oncol (2021) 27:100353. doi: 10.1016/j.jbo.2021.100353 33889483 PMC8047448

[41] Sun M Trinh QD. A Surveillance, Epidemiology and End Results (SEER) Database Malfunction: Perceptions, Pitfalls and Verities. BJU Int (2016) 117:551–2. doi: 10.1111/bju.13226 26190064

