# Supplementary figures and images for: Impact of Limb Salvage on Prognosis of Patients Diagnosed With Extremity Bone and Soft Tissue Sarcomas
Source: Front Oncol. 2022 Jun 6;12:873323. doi: 10.3389/fonc.2022.873323 (PMC9208618; doi:10.3389/fonc.2022.873323)

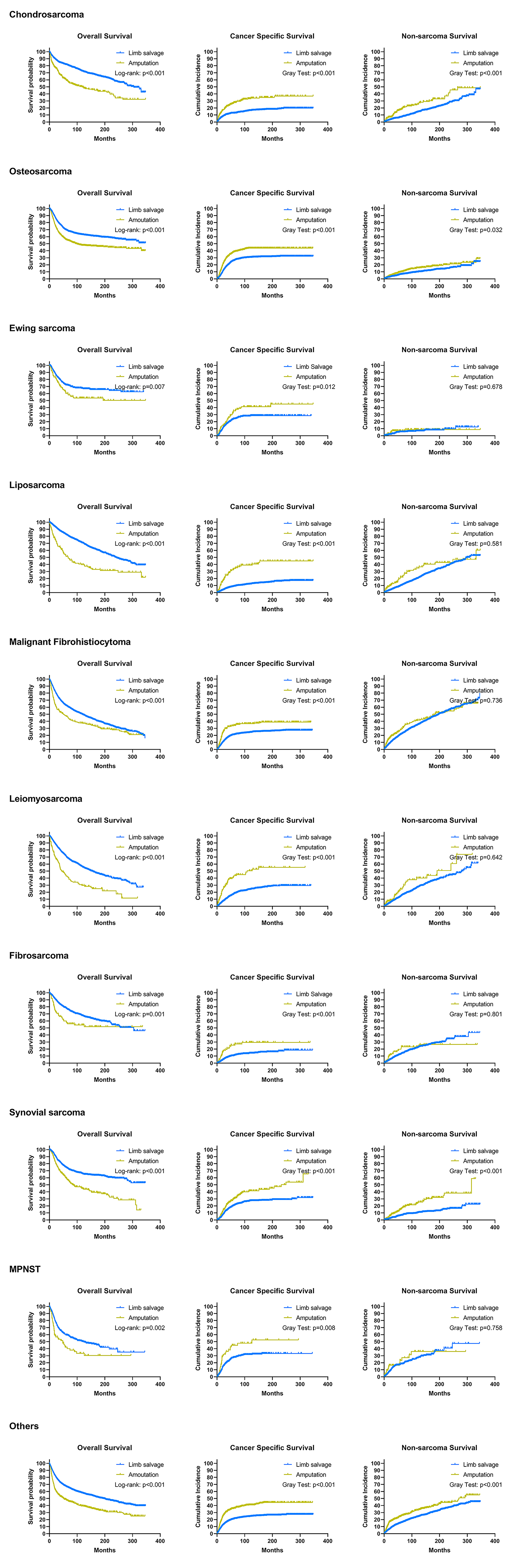

Supplement: Supplementary Figure 1 — Subgroup analysis: the graph showed Kaplan-Meier curves of overall survival, and cumulative incidence curves of death resulting from sarcomas and death resulting from other non-sarcomas causes. MFH, malignant fibro histiocytoma; MPNST, malignant peripheral nerve sheath tumors. [file Image_1.tif]

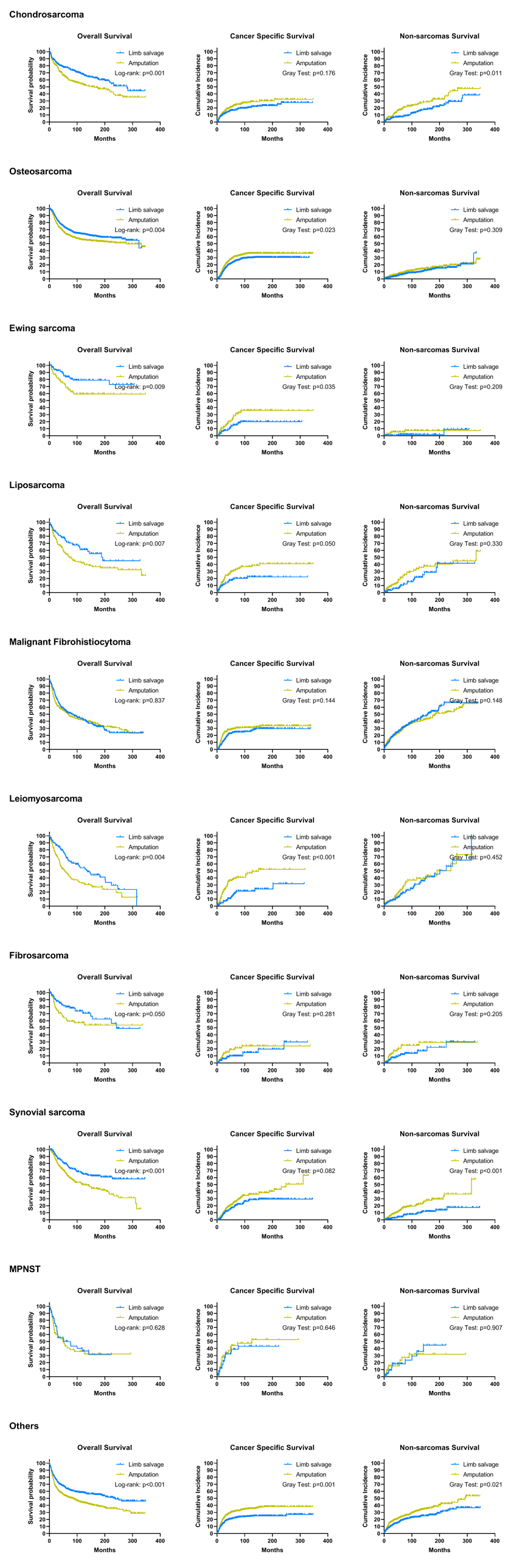

Supplement: Supplementary Figure 2 — Using subgroup with localized and regional sarcomas in the cohort after PSM, the graph showed Kaplan-Meier curves of overall survival, and cumulative incidence curves of death resulting from sarcomas and death resulting from other non-sarcomas causes. MFH, malignant fibro histiocytoma; MPNST, malignant peripheral nerve sheath tumors. [file Image_2.tif]

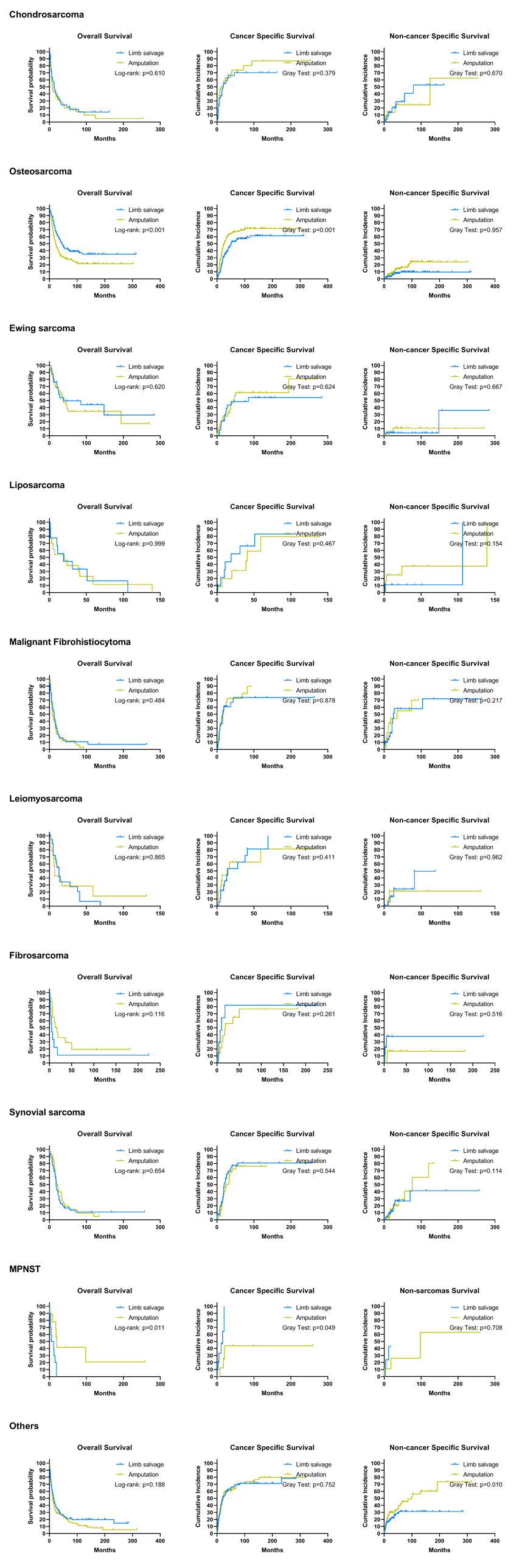

Supplement: Supplementary Figure 3 — Using subgroup with distant metastases in the cohort after PSM, the graph showed Kaplan-Meier curves of overall survival, and cumulative incidence curves of death resulting from sarcomas and death resulting from other non-sarcomas causes. MFH, malignant fibro histiocytoma; MPNST, malignant peripheral nerve sheath tumors. [file Image_3.tif]
