# Supplementary material for: Impact of Limb Salvage on Prognosis of Patients Diagnosed With Extremity Bone and Soft Tissue Sarcomas
Source: Front Oncol. 2022 Jun 6;12:873323. doi: 10.3389/fonc.2022.873323 (PMC9208618; doi:10.3389/fonc.2022.873323)
Supplement: Supplementary file 4 [file Table_1.docx]

**Supplementary Table 1. Logistic and Cox proportional hazards regression analyses of different variables considered for OS and CSS for patients with extremity bone and soft tissue sarcomas.**

|  | **Logistic regression model** | | | |  | **Cox proportional hazards model** | | | |
| --- | --- | --- | --- | --- | --- | --- | --- | --- | --- |
| **Overall Survival** | | **Cancer Specific Survival** | |  | **Overall Survival** | | **Cancer Specific Survival** | |  |
|  | **OR (95%CI)** | **P value** | **OR (95%CI)** | **P value** |  | **HR (95%CI)** | **P value** | **HR (95%CI)** | **P value** |
| **Surgery** |  |  |  |  |  |  |  |  |  |
| Amputation | - |  | - |  |  | - |  | - |  |
| Limb salvage | 0.38 (0.29,0.49) | <0.001 | 0.65 (0.59,0.71) | <0.001 |  | 0.70 (0.66,0.74) | <0.001 | 0.67 (0.62,0.72) | <0.001 |
| **Age at diagnosis** | 1.06 (1.06,1.06) | <0.001 | 1.02 (1.01,1.03) | <0.001 |  | 1.04 (1.03,1.05) | <0.001 | 1.02 (1.01,1.03) | <0.001 |
| **Sex** |  |  |  |  |  |  |  |  |  |
| Female | - |  | - |  |  | - |  | - |  |
| Male | 1.43 (1.35,1.52) | <0.001 | 1.42 (1.16,1.32) | <0.001 |  | 1.28 (1.23,1.33) | <0.001 | 1.21 (1.15,1.28) | <0.001 |
| **Race** |  |  |  |  |  |  |  |  |  |
| White | - |  | - |  |  | - |  | - |  |
| Black | 1.18 (1.08,1.30) | <0.001 | 1.18 (1.07,1.30) | 0.001 |  | 1.14 (1.07,1.21) | <0.001 | 1.15 (1.06,1.25) | 0.001 |
| Other | 0.84 (0.76,0.94) | 0.001 | 0.88 (0.78,0.99) | 0.030 |  | 0.92 (0.86,0.99) | 0.022 | 0.91 (0.82,1.01) | 0.066 |
| **Year of diagnosis** |  |  |  |  |  |  |  |  |  |
| 1988-1999 | - |  | - |  |  | - |  | - |  |
| 2000-2009 | 0.46 (0.42,0.49) | <0.001 | 0.81 (0.74,0.88) | <0.001 |  | 0.92 (0.87,0.96) | <0.001 | 0.91 (0.85,0.97) | 0.005 |
| 2010-2017 | 0.13 (0.11,0.14) | <0.001 | 0.38 (0.33,0.42) | <0.001 |  | 0.86 (0.80,0.93) | <0.001 | 0.83 (0.75,0.92) | 0.000 |
| **Marital status** |  |  |  |  |  |  |  |  |  |
| Married | - |  | - |  |  | - |  | - |  |
| Unmarried | 1.70 (1.60,1.81) | <0.001 | 1.17 (1.09,1.25) | <0.001 |  | 1.42 (1.36,1.48) | <0.001 | 1.24 (1.17,1.31) | <0.001 |
| Unknow | 1.12 (0.97,1.30) | 0.111 | 0.96 (0.80,1.14) | 0.639 |  | 1.09 (0.99,1.20) | 0.089 | 0.97 (0.83,1.13) | 0.707 |
| **Education** |  |  |  |  |  |  |  |  |  |
| High | - |  | - |  |  | - |  | - |  |
| Median | 1.10 (1.02,1.19) | 0.018 | 1.15 (1.05,1.25) | 0.002 |  | 1.01 (0.96,1.07) | 0.622 | 1.08 (1.10,1.16) | 0.033 |
| Low | 1.06 (0.98,1.16) | 0.151 | 1.07 (0.97,1.17) | 0.188 |  | 1.00 (0.94,1.05) | 0.894 | 1.02 (0.95,1.11) | 0.544 |
| **Income** |  |  |  |  |  |  |  |  |  |
| High | - |  | - |  |  | - |  | - |  |
| Median | 1.09 (1.02,1.18) | 0.015 | 1.02 (0.94,1.10) | 0.660 |  | 1.04 (1.00,1.10) | 0.072 | 1.02 (0.95,1.09) | 0.615 |
| Low | 1.15 (1.06,1.25) | 0.001 | 1.01 (0.92,1.10) | 0.859 |  | 1.09 (1.03,1.15) | 0.001 | 1.03 (0.96,1.11) | 0.457 |
| **Insurance** |  |  |  |  |  |  |  |  |  |
| Insured | - |  | - |  |  | - |  | - |  |
| Any Medicaid | 1.46 (1.29,1.65) | <0.001 | 1.05 (0.91,1.20) | 0.534 |  | 1.37 (1.25,1.50) | <0.001 | 1.13 (1.01,1.27) | 0.039 |
| Uninsured | 1.67 (1.30,2.12) | <0.001 | 1.42 (1.09,1.83) | 0.009 |  | 1.55 (1.30,1.80) | <0.001 | 1.45 (1.17,1.80) | 0.001 |
| Unknown | 1.47 (1.36,1.59) | <0.001 | 1.13 (1.03,1.24) | 0.007 |  | 1.10 (1.04,1.16) | 0.001 | 1.06 (0.98,1.14) | 0.152 |
| **Location** |  |  |  |  |  |  |  |  |  |
| Upper Limb | - |  | - |  |  | - |  | - |  |
| Lower Limb | 1.18 (1.10,1.26) | <0.001 | 1.36 (1.26,1.46) | <0.001 |  | 1.11 (1.07,1.16) | <0.001 | 1.26 (1.18,1.34) | <0.001 |
| **Laterality** |  |  |  |  |  |  |  |  |  |
| Left | - |  | - |  |  | - |  | - |  |
| Right | 0.96 (0.91,1.02) | 0.161 | 0.98 (0.92,1.04) | 0.538 |  | 0.98 (0.95,1.02) | 0.400 | 0.98 (0.93,1.03) | 0.395 |
| Others | 3.06 (1.40,7.07) | 0.007 | 3.43 (1.72,6.81) | 0.000 |  | 1.82 (1.27,2.62) | 0.001 | 2.63 (1.69,4.09) | <0.001 |
| **Grade** |  |  |  |  |  |  |  |  |  |
| Grade I | - |  | - |  |  | - |  | - |  |
| Grade II | 1.52 (1.35,1.71) | <0.001 | 2.60 (2.15,3.16) | <0.001 |  | 1.43 (1.31,1.57) | <0.001 | 2.68 (2.24,3.22) | <0.001 |
| Grade III | 2.88 (2.55,3.25) | <0.001 | 5.76 (4.81,6.94) | <0.001 |  | 2.45 (2.25,2.67) | <0.001 | 5.96 (5.02,7.08) | <0.001 |
| Grade IV | 2.73 (2.43,3.07) | <0.001 | 5.93 (4.96,7.13) | <0.001 |  | 2.41 (2.21,2.62) | <0.001 | 6.12 (5.16,7.27) | <0.001 |
| Unknown | 2.11 (1.88,2.36) | <0.001 | 3.57 (2.99,4.30) | <0.001 |  | 1.84 (1.69,2.00) | <0.001 | 3.83 (3.23,4.55) | <0.001 |
| **Stage** |  |  |  |  |  |  |  |  |  |
| Localized | - |  | - |  |  | - |  | - |  |
| Regional | 1.61 (1.51,1.73) | <0.001 | 1.78 (1.66,1.92) | <0.001 |  | 1.42 (1.35,1.48) | <0.001 | 1.74 (1.63,1.85) | <0.001 |
| Distant | 8.21 (7.25,9.30) | <0.001 | 5.71 (5.14,6.34) | <0.001 |  | 4.56 (4.29,4.84) | <0.001 | 5.55 (5.15,5.99) | <0.001 |
| Unknow | 1.29 (1.12,1.49) | <0.001 | 1.41 (1.20,1.66) | <0.001 |  | 1.43 (1.29,1.57) | <0.001 | 1.78 (1.55,2.05) | <0.001 |
| **Histology** |  |  |  |  |  |  |  |  |  |
| Chondrosarcoma | - |  | - |  |  | - |  | - |  |
| Osteosarcoma | 1.41 (1.21,1.65) | <0.001 | 0.99 (0.83,1.18) | 0.888 |  | 1.33 (1.20,1.47) | <0.001 | 1.02 (0.88,1.18) | 0.803 |
| Ewing sarcoma | 1.46 (1.16,1.83) | 0.001 | 0.83 (0.70,0.98) | 0.027 |  | 1.47 (1.25,1.72) | <0.001 | 0.90 (0.71,1.03) | 0.141 |
| Liposarcoma | 0.88 (0.77,1.00) | 0.056 | 0.52 (0.45,0.61) | <0.001 |  | 0.89 (0.81,0.98) | 0.013 | 0.59 (0.52,0.67) | <0.001 |
| MFH | 1.01 (0.88,1.15) | 0.928 | 0.78 (0.66,0.93) | 0.005 |  | 0.93 (0.85,1.01) | 0.088 | 0.74 (0.64,0.85) | <0.001 |
| Leiomyosarcoma | 0.99 (0.86,1.15) | 0.926 | 0.58 (0.49,0.68) | <0.001 |  | 0.93 (0.84,1.02) | 0.130 | 0.62 (0.55,0.72) | <0.001 |
| Fibrosarcoma | 0.70 (0.59,0.82) | <0.001 | 0.87 (0.69,1.11) | 0.263 |  | 0.78 (0.70,0.88) | <0.001 | 0.99 (0.82,1.19) | 0.880 |
| Synovial sarcoma | 1.41 (1.19,1.66) | <0.001 | 0.71 (0.61,0.82) | <0.001 |  | 1.29 (1.15,1.44) | <0.001 | 0.83 (0.74,0.93) | 0.002 |
| MPNST | 2.21 (1.78,2.73) | <0.001 | 0.48 (0.40,0.59) | <0.001 |  | 1.73 (1.51,1.98) | <0.001 | 0.53 (0.44,0.63) | <0.001 |
| Others | 1.04 (0.92,1.18) | 0.534 | 1.16 (0.92,1.46) | 0.200 |  | 1.11 (1.02,1.21) | 0.017 | 1.29 (1.08,1.55) | 0.006 |
| **Radiation** |  |  |  |  |  |  |  |  |  |
| No/Unknown | - |  | - |  |  | - |  | - |  |
| Yes | 1.09 (1.02,1.17) | 0.008 | 1.42 (1.32,1.53) | 0.000 |  | 0.98 (0.94,1.03) | 0.439 | 1.20 (1.13,1.28) | 0.000 |
| **Chemotherapy** |  |  |  |  |  |  |  |  |  |
| No/Unknown | - |  | - |  |  | - |  | - |  |
| Yes | 2.03 (1.87,2.20) | 0.000 | 1.98 (1.83,2.16) | 0.000 |  | 1.42 (1.35,1.50) | 0.000 | 1.60 (1.49,1.71) | 0.000 |

Abbreviations: OR, odds ratios; HR, hazard ratios; MFH, malignant fibrous histiocytoma; MPNST, malignant peripheral nerve sheath tumor.
