# Supplementary material for: Impact of Limb Salvage on Prognosis of Patients Diagnosed With Extremity Bone and Soft Tissue Sarcomas
Source: Front Oncol. 2022 Jun 6;12:873323. doi: 10.3389/fonc.2022.873323 (PMC9208618; doi:10.3389/fonc.2022.873323)
Supplement: Supplementary file 5 [file Table_2.docx]

**Supplementary Table 2. The non-cancer specific causes of death among patients with extremity bone and soft tissue sarcomas.**

| Causes of Death | number | morbidity |
| --- | --- | --- |
| Cardiovascular diseases | 1463 | 26% |
| Other Cause of Death | 618 | 11% |
| Miscellaneous Malignant Cancer | 610 | 11% |
| Lung and Bronchus | 571 | 10% |
| COPD and Allied Cond | 201 | 4% |
| State DC not available or state DC available | 190 | 3% |
| Accidents and Adverse Effects | 142 | 3% |
| Non-Melanoma Skin | 124 | 2% |
| Alzheimer diseases | 120 | 2% |
| In situ, benign or unknown behavior neoplasm | 117 | 2% |
| Infection | 220 | 4% |
| Diabetes Mellitus | 108 | 2% |
| Colon excluding Rectum | 95 | 2% |
| Breast | 95 | 2% |
| Prostate | 92 | 2% |
| Non-Hodgkin Lymphoma | 79 | 1% |
| Pancreas | 72 | 1% |
| Melanoma of the Skin | 69 | 1% |
| Symptoms, Signs and Ill-Defined Conditions | 56 | 1% |
| Acute Myeloid Leukemia | 54 | 1% |
| Nephritis, Nephrotic Syndrome and Nephrosis | 53 | 1% |
| Hypertension without Heart Disease | 52 | 1% |
| Suicide and Self-Inflicted Injury | 50 | 1% |
| Brain and Other Nervous System | 46 | 1% |
| Stomach | 38 | 1% |
| Urinary Bladder | 31 | 1% |
| Chronic Liver Disease and Cirrhosis | 29 | 1% |
| Others | 249 | 4% |
| ALL | 5644 | 100% |

Abbreviations: COPD, chronic obstructive pulmonary diseases
