# Supplementary material for: Impact of Limb Salvage on Prognosis of Patients Diagnosed With Extremity Bone and Soft Tissue Sarcomas
Source: Front Oncol. 2022 Jun 6;12:873323. doi: 10.3389/fonc.2022.873323 (PMC9208618; doi:10.3389/fonc.2022.873323)
Supplement: Supplementary file 6 [file Table_3.docx]

**Supplementary Table 3. The risk of dying from some non-cancer diseases among patients with extremity bone and soft tissue sarcomas.**

|  | **CVD** | **COPD** | **Accident** | **Infection** | **DM** | **Suicide** | **AZ** | **ND** |
| --- | --- | --- | --- | --- | --- | --- | --- | --- |
| **Chondrosarcoma** |  |  |  |  |  |  |  |  |
| Number of deaths | 90 | 18 | 15 | 10 | 9 | 6 | 4 | 1 |
| Mortality | 439.26 | 87.85 | 73.21 | 48.81 | 43.93 | 29.28 | 35.04 | 4.88 |
| SMR | 1.36 | 1.78 | 1.77 | 1.85 | 1.55 | 1.95 | 1.40 | 0.37 |
| 95%CI | 1.11 to 1.67 | 1.12 to 2.82 | 1.07 to 2.94 | 0.99 to 3.43 | 0.81 to 2.99 | 0.87 to 4.33 | 0.53 to 3.73 | 0.05 to 2.61 |
| **Osteosarcoma** |  |  |  |  |  |  |  |  |
| Number of deaths | 31 | 3 | 11 | 11 | 1 | 8 | 2 | 1 |
| Mortality | 120.39 | 11.65 | 42.72 | 42.72 | 3.88 | 31.07 | 7.77 | 3.88 |
| SMR | 1.92 | 1.28 | 1.62 | 5.61 | 0.65 | 3.69 | 2.78 | 1.35 |
| 95%CI | 1.35 to 2.74 | 0.41 to 3.98 | 0.90 to 2.92 | 3.11 to 10.14 | 0.09 to 4.63 | 1.84 to 7.37 | 0.69 to 11.11 | 0.19 to 9.57 |
| **Ewing sarcoma** |  |  |  |  |  |  |  |  |
| Number of deaths | 2 | 1 | 1 | 3 | 0 | 1 | 0 | 0 |
| Mortality | 36.99 | 18.50 | 18.50 | 55.49 | 0.00 | 18.50 | 0.00 | 0.00 |
| SMR | 1.94 | 6.60 | 0.77 | 13.50 | 0.00 | 2.44 | 0.00 | 0.00 |
| 95%CI | 0.49 to 7.78 | 0.93 to 46.84 | 0.11 to 5.46 | 4.35 to 41.84 | 0.00 | 0.34 to 17.32 | 0.00 | 0.00 |
| **Liposarcoma** |  |  |  |  |  |  |  |  |
| Number of deaths | 303 | 33 | 27 | 32 | 18 | 7 | 36 | 12 |
| Mortality | 750.04 | 81.69 | 66.84 | 79.21 | 44.56 | 17.33 | 89.11 | 29.70 |
| SMR | 1.36 | 0.97 | 1.31 | 2.08 | 0.98 | 1.04 | 2.99 | 1.21 |
| 95%CI | 1.22 to 1.53 | 0.69 to 1.37 | 0.90 to 1.91 | 1.47 to 2.95 | 0.62 to 1.56 | 0.50 to 2.19 | 2.15 to 4.14 | 0.69 to 2.14 |
| **MFH** |  |  |  |  |  |  |  |  |
| Number of deaths | 421 | 62 | 31 | 53 | 31 | 8 | 38 | 14 |
| Mortality | 1266.37 | 186.50 | 93.25 | 159.42 | 93.25 | 24.06 | 114.30 | 42.11 |
| SMR | 1.51 | 1.54 | 1.66 | 3.52 | 1.52 | 1.48 | 2.60 | 1.26 |
| 95%CI | 1.38 to 1.67 | 1.20 to 1.98 | 1.17 to 2.36 | 2.69 to 4.60 | 1.07 to 2.17 | 0.74 to 2.97 | 1.89 to 3.57 | 0.74 to 2.12 |
| **Leiomyosarcoma** |  |  |  |  |  |  |  |  |
| Number of deaths | 145 | 27 | 8 | 229 | 8 | 4 | 13 | 7 |
| Mortality | 837.16 | 155.88 | 46.19 | 167.43 | 46.19 | 23.09 | 75.06 | 40.41 |
| SMR | 1.23 | 1.49 | 0.81 | 3.92 | 0.88 | 1.30 | 1.69 | 1.32 |
| 95%CI | 1.05 to 1.45 | 1.02 to 2.17 | 0.41 to 1.62 | 2.72 to 5.64 | 0.44 to 1.75 | 0.49 to 3.46 | 0.98 to 2.91 | 0.63 to 2.77 |
| **Fibrosarcoma** |  |  |  |  |  |  |  |  |
| Number of deaths | 79 | 12 | 12 | 17 | 5 | 5 | 7 | 3 |
| Mortality | 627.15 | 95.26 | 95.26 | 134.96 | 39.69 | 39.69 | 55.57 | 23.82 |
| SMR | 1.07 | 1.01 | 1.76 | 3.31 | 0.84 | 2.70 | 1.17 | 0.81 |
| 95%CI | 0.86 to 1.34 | 0.57 to 1.78 | 1.00 to 3.09 | 2.06 to 5.32 | 0.35 to 2.02 | 1.12 to 6.48 | 0.56 to 2.46 | 0.26 to 2.50 |
| **Synovial sarcoma** |  |  |  |  |  |  |  |  |
| Number of deaths | 21 | 3 | 5 | 4 | 3 | 2 | 0 | 0 |
| Mortality | 147.19 | 21.03 | 35.04 | 28.04 | 21.03 | 14.02 | 0.00 | 0.00 |
| SMR | 1.36 | 1.29 | 1.06 | 2.06 | 1.92 | 1.18 | 0.00 | 0.00 |
| 95%CI | 0.89 to 2.09 | 0.42 to 4.01 | 0.44 to 2.55 | 0.77 to 5.49 | 0.62 to 5.96 | 0.29 to 4.72 | 0.00 | 0.00 |
| **MPNST** |  |  |  |  |  |  |  |  |
| Number of deaths | 24 | 0 | 1 | 8 | 3 | 0 | 0 | 3 |
| Mortality | 598.75 | 0.00 | 24.95 | 199.58 | 74.84 | 0.00 | 0.00 | 74.84 |
| SMR | 1.78 | 0.00 | 0.55 | 7.01 | 2.53 | 0.00 | 0.00 | 4.17 |
| 95%CI | 1.19 to 2.65 | 0.00 | 0.08 to 3.89 | 3.51 to 14.02 | 0.82 to 7.85 | 0.00 | 0.00 | 1.34 to 12.92 |
| **Others** |  |  |  |  |  |  |  |  |
| Number of deaths | 329 | 42 | 31 | 53 | 30 | 9 | 20 | 12 |
| Mortality | 734.48 | 93.76 | 69.21 | 118.32 | 66.97 | 20.09 | 44.65 | 26.79 |
| SMR | 1.58 | 1.33 | 1.44 | 3.57 | 1.81 | 1.43 | 1.28 | 1.21 |
| 95%CI | 1.42 to 1.76 | 0.98 to 1.80 | 1.01 to 2.04 | 2.73 to 4.67 | 1.27 to 2.60 | 0.75 to 2.75 | 0.83 to 1.98 | 0.69 to 2.14 |
| **All** |  |  |  |  |  |  |  |  |
| Number of deaths | 1445 | 201 | 142 | 220 | 108 | 50 | 120 | 53 |
| Mortality | 662.01 | 92.09 | 65.06 | 100.79 | 49.48 | 22.91 | 54.98 | 24.28 |
| SMR | 1.43 | 1.32 | 1.41 | 3.21 | 1.34 | 1.60 | 1.96 | 1.18 |
| 95%CI | 1.36 to 1.50 | 1.15 to 1.51 | 1.20 to 1.66 | 2.81 to 3.67 | 1.11 to 1.62 | 1.21 to 2.11 | 1.64 to 2.35 | 0.90 to 1.55 |

Abbreviations: CVD, cardiovascular diseases; COPD, chronic obstructive pulmonary diseases; SMR, standardized mortality ratio; CI, confidence intervals; MFH, malignant fibrous histiocytoma; MPNST, malignant peripheral nerve sheath tumor.
